# Characterization of the microbiome associated with in situ earthen materials

**DOI:** 10.1186/s40793-019-0350-6

**Published:** 2020-01-20

**Authors:** Alexis Simons, Alexandra Bertron, Jean-Emmanuel Aubert, Christophe Roux, Christine Roques

**Affiliations:** 1 Laboratoire de Génie Chimique, Université de Toulouse, UMR 5503 UPS – CNRS – INPT, Toulouse, France; 2 Laboratoire Matériaux et Durabilité des Constructions, Université de Toulouse, UPS - INSA, Toulouse, France; 3 Laboratoire de Recherche en Sciences Végétales, Université de Toulouse, UMR 5546 UPS – CNRS, Castanet-Tolosan, France

**Keywords:** Earthen building materials, High-throughput sequencing, Microbial diversity, Built-environment microbiome, Bacterial communities, Fungal communities

## Abstract

**Background:**

The current increase in public awareness of environmental risks is giving rise to a growth of interest in the microbiological safety of buildings. In particular, microbial proliferation on construction materials can be responsible for the degradation of indoor air quality that can increase health-risk to occupants. Raw earth materials are still widely used throughout the world and, in some cases, are linked to heritage habitats, as in the southwest of France. Moreover, these building materials are currently the subject of renewed interest for ecological and economic reasons. However, the microbial status of earthen materials raises major concerns: could the microbiome associated with such natural materials cause disease in building occupants? Very few analyses have been performed on the microbial communities present on these supports. Characterizing the raw earth material microbiome is also important for a better evaluation and understanding of the susceptibility of such materials to microbial development. This study presents the distribution of in situ bacterial and fungal communities on different raw earth materials used in construction. Various buildings were sampled in France and the microbial communities present were characterized by amplicon high-throughput sequencing (bacterial 16S rRNA gene and fungal ITS1 region). Bacterial culture isolates were identified at the species level by MALDI-TOF mass spectrometry.

**Results:**

The major fungal and bacterial genera identified were mainly associated with conventional outdoor and indoor environmental communities, and no specific harmful bacterial species were detected on earthen materials. However, contrary to expectations, few human-associated genera were detected in dwellings. We found lower microbial alpha-diversity in earthen material than is usually found in soil, suggesting a loss of diversity during the use of these materials in buildings. Interestingly enough, the main features influencing microbial communities were building history and room use, rather than material composition.

**Conclusions:**

These results constitute a first in-depth analysis of microbial communities present on earthen materials in situ and may be considered as a first referential to investigate microbial communities on such materials according to environmental conditions and their potential health impact. The bacterial and fungal flora detected were similar to those found in conventional habitats and are thought to be mainly impacted by specific events in the building’s life, such as water damage.

## Background

Earthen building materials have been widely used throughout the world, and continue to be used in many countries, with nearly a third of the world’s population living in this type of construction [1]. Raw earth is mainly used in Africa, China and the Middle East, but there are also many heritage habitats throughout Europe, including in certain regions of France [2]. The environmental impact of building materials is an important issue and is becoming the object of renewed interest in connection with ecological challenges. In addition to heritage matters, earthen materials present several putative solutions to tackle these environmental issues: they promote a natural regulation of the indoor humidity [3] and their ecological impact [4] and cost [5] are low. Earthen materials can also contain plant fibres and aggregates (straw, hemp, etc.), which enhance the thermal insulation and decrease the risk of cracking during the drying step of the manufacture of the material [6]. However, the addition of cellulose and/or lignin may induce greater sensitivity to fungal proliferation [7]. Few proliferation issues have been reported on earthen materials but some visible mould developments have been detected in certain situations (water-related accident, poor ventilation during the few first days of drying, etc.) [8, 9]. These observations could raise questions about the conditions of fungal development on earthen materials.

The problems linked to the indoor air quality and microbial proliferation are indeed very important, with 10 to 50% of buildings contaminated by moulds in Europe, North America, Australia, India and Japan [10]. Humans spend more than 80% of their time inside buildings [11], and so are strongly exposed to the indoor air [12]. Under specific environmental conditions i.e. high humidity [13, 14], microorganisms are highly likely to develop on conventional building materials. The microbial growth within dwellings leads to a release of harmful particles (volatile organic compounds, toxins, parietal compounds, spores, etc.) in the indoor air and may induce health issues for the occupants, from asthma and allergy to ocular and pulmonary irritations and infections [15]. The identification of microorganisms present on the surface of construction materials in buildings is an important challenge that must be taken up if the risks of proliferation are to be better apprehended.

The microbial flora composition of conventional constructions was first characterized by cultural approaches. Studies on indoor air and dust microflora have shown the major presence of *Ascomycota* and, more precisely, the genera *Cladosporium*, *Aspergillus* and *Penicillium* [16, 17], for the fungal communities, and *Micrococcus, Staphylococcus* and *Bacillus* for the bacterial ones [18, 19]. The culturable part of microbial diversity is estimated at a very low percentage, however (1%) [20], which depends on the environment or the microorganisms studied, with, for example, a lower frequency of cultivability for *Basidiomycota* [21] or Gram negative bacteria [22].

The use of DNA and, more specifically, of metabarcoding methods, to study microbial ecology has deepened the observable diversity [23, 24]. Molecular barcodes, such as different regions of the 16S rRNA gene for bacteria or internal transcribed spacer (ITS) regions for fungi, allow a great microbial diversity to be detected in indoor environments [25–27]. Bacterial and fungal indoor communities have been characterized for various types of building, such as dwellings [26, 28, 29], university classrooms [30] or childcare facilities [31], highlighting the main factors that drive microbial composition in buildings. For instance, Adams and co-authors [32] investigated the microbiome of indoor surfaces across standardized residences and revealed the major impact of the outdoor fungal flora on the indoor ones, rather than the influence of the room sampled or the human activities. This predominance of outdoor fungi in the buildings, in particular for the indoor air, has been confirmed in other studies [31]. Major fungal taxa detected by metabarcoding in buildings are related to *Ascomycota* (i.e. *Cladosporium*, *Aspergillus*, *Penicillium*, *Alternaria*, *Fusarium*, *Aureobasidium*, *Epicoccum*, *Phoma*) and *Basidiomycota* (i.e. *Cryptococcus*, *Wallemia*) [25, 28, 29, 31–34]. Variations in indoor air and dust fungal microbiome may be also observed with respect to various factors such as geographical location [32, 34], building environment (urban/rural) [35], or seasons [32, 36]. In contrast, outdoor bacterial flora have a very little impact on the bacterial communities detected in indoor air, dust and surfaces, which are essentially linked to bacteria associated with humans (i.e. genera *Micrococcus*, *Staphylococcus*, *Propionibacterium*, *Streptococcus*) [26, 30, 37, 38]. By comparing the bacterial flora of surfaces in several rooms with the microbiome of their inhabitants, Lax et al. [27] showed that the floras were more similar within a single house than between two rooms with the same function in two different houses. Studies on bacterial and fungal communities of conventional buildings and construction materials, such as plaster and concrete, are increasing, which is not the case for earth-based construction materials.

Although raw earth is still used for construction in the present day, few data exist on the microbial flora associated with this material. Our study thus aimed to provide a first overview of the microorganisms present in situ on earthen construction materials in France. Our objectives were i) to characterize and make an inventory of the microorganisms present on earthen materials used in buildings according to their location and use, and ii) to evaluate the influence of additional plant fibres in earthen materials, or of room use, on microbial communities. This study will provide better knowledge of the microorganisms present and probably of those able to proliferate on earthen materials, as well as highlighting the impact of certain factors on the composition of these bacterial and fungal communities.

## Methods

### Study sites and sample collection

Volunteer building owners were contacted for this study. A total of 12 buildings with earthen walls were included (See Additional file 1: Table S1), resulting in 76 samples of earthen materials. Three different sampling campaigns were carried out: from October to November 2015, in July 2016 and from October 2016 to January 2017. The buildings were located in the south-west (9 sites) and east of France (3 sites). Four combinations of material composition and location conditions were set up as follows: i) earthen materials without vegetal aggregates – 6 dwelling sites, noted E-D; ii) earthen materials with vegetal aggregates – 5 dwelling sites, noted V-D; iii) earthen materials without vegetal aggregates – 3 non-dwelling sites, noted E-ND; iv) earthen materials with vegetal aggregates – 3 non-dwelling sites, noted V-ND. All materials sampled were in normal moisture conditions at the time of sampling (i.e. no visible proliferation due to water accumulation, air relative humidity (RH) between 50 and 65%), and the only site with previous mould proliferation less than 1 year before sampling had returned to a dry state without visible mould a few months previously.

Samples on the surface of earthen walls were collected using sterile scalpels. Small areas (4 cm^2^) were scratched (sampling areas were located at different heights if possible i.e. 40 cm to 190 cm from the floor), in order to obtain 2 to 3 g of material per area (dwellings - hall, kitchen, bedroom: 4 and 6 areas per wall; non-dwellings - barn, cellar: 1 to 2 areas per wall). Part of the sampled materials from 5 sites was used for cultural isolation, while samples from all sites were stored at − 80 °C until DNA extraction.

### Cultural isolation and identification

Microbial sampling on earthen materials had been optimized previously [39]. Briefly, 1 g of sampled materials was mixed with 10 mL of recovery medium (PBS + 5% Tween 80) and shaken at 300 rpm for 30 min (MaxQ 4000 Agitator - Thermo Scientific, Waltham - Massachusetts, U.S.A.). Suspensions were diluted, deposited on culture media and incubated at different temperatures (bacteria: Tryptone Soy Agar (TSA) – 30 °C). Identifications of bacterial isolates were carried out using MALDI-TOF mass spectrometry, which is currently used for the identification of clinical and environmental strains [40, 41]. A sample of a bacterial colony was homogeneously deposited on a MALDI target position (MSP 96 polished steel BC target). The deposit was then coated with 1 μL of 70% formic acid [42]. After drying, 1 μL of IVD HCCA matrix (Bruker, Billerica - Massachusetts, U.S.A.), reconstituted with 250 μL organic solvent solution (acetonitrile 50%, water 47.5%, TFA 2.5%) (Sigma-Aldrich, Saint-Louis - Missouri, U.S.A.), was deposited on its surface and left to dry. The internal control BTS (Bacterial Test Standard; *E. coli*, Bruker) was added on a MALDI target position. The protein profiles of each spot were analysed by MALDI-TOF BioTyper (Bruker, Billerica - Massachusetts, U.S.A.). The acquired spectrum was compared with the reference spectra contained in the general database IVD MALDI BioTyper, version 4.0(5627). Identification results were accepted when the spectrum congruence score threshold was greater than or equal to 1.75.

### DNA extraction, PCR and sequencing

Genomic DNA extraction was performed on 250 mg of material with the ZymoBIOMICS DNA Miniprep kit (Zymo Research, Irvine – California, U.S.A.) according to the manufacturer’s instructions. For PCR amplification, the primers used for bacteria were 343F (5′-ACGGRAGGCAGCAG-3′ [43]) and 784R (5′-TACCAGGGTATCTAATCCT-3′ [44]) targeting the region V3 – V4 of the 16S rRNA genes. For fungi, the internal transcribed spacer (ITS) region 1 was targeted with primers (5′-CTTGGTCATTTAGAGGAAGTAA-3′ [45] and 5′-GCTGCGTTCTTCATCGATGC-3′ [46]). PCR amplification was carried out with the MTP Taq DNA polymerase (Sigma-Aldrich, Saint-Louis - Missouri, U.S.A.) under the following conditions: 5 min at 94 °C; 35 cycles of 30 s at 94 °C, 1 min at 55 °C and 30 s at 72 °C; and 7 min at 72 °C. The efficiency of PCR amplifications was checked by electrophoresis on agarose gel. In case of an absence of amplification, probably due to the presence of polymerase inhibitors in extracts (such as humic acids or clay particles), dilution of one tenth to one thousandth of DNA extract restored amplification. Amplicons were purified on Clean PCR beads (MokaScience, La Madeleine, France) and their quality was checked with a Fragment Analyzer (Advanced Analytical Technologies, Ankeny – Iowa, USA). All samples were processed at the GeT-PlaGe platform (Auzeville, France) for second PCR with Illumina Miseq primers containing indexes, equimolar library preparation and Illumina MiSeq 2 × 250 bp (San Diego – California, U.S.A.) using the standard protocol.

### Bioinformatics analysis

The following bioinformatic pipeline was used for both types of amplicons. Raw reads were assembled with a minimal overlap of 10 bp using the MOTHUR v.1.35.1 software [47]. Assembled reads with a length of less than 100 bp were discarded. Detection of chimera was performed with UPARSE, implemented in USEARCH v10.0.240 [48]. Remaining reads were clustered at 0.97. In order to keep only the ITS region, the border regions 5.8S in 5′ and 28S in 3′ were removed using the Hidden Markov Model (HMM) [49]. Operational taxonomic unit (OTU) sequences were assigned with the USEARCH global command based on the UNITE database [50] for ITS1 reads and on the RDP [51] database for 16S rRNA gene ones. Singleton OTUs were removed. Unassigned OTUs containing 50 reads or more were re-aligned with the non-redundant GenBank database with BLAST 2.7.0 [52]. ITS1 libraries contained 3,231,180 reads, split among 3654 OTUs, and 16S rRNA gene libraries contained 2,116,341 reads forming 14,080 OTUs.

### Community analyses

Statistical analysis was performed using R v3.4.1. Each library was normalized by rarefaction to the smallest number of reads across all libraries for each region. After normalization, a total of 2,085,468 reads for ITS1 sequences and 761,775 reads for 16S rRNA gene sequences were contained in 3544 and 13,174 OTUs, respectively. The α-diversity was calculated for each sample using the Shannon index (log e base) [53], and site index means were compared with a Mann-Whitney test. Fungal and bacterial Shannon indices were compared with the Spearman correlation test. The β-diversity was calculated regarding the mean relative OTU abundance by site and a Bray-Curtis distance matrix [54] was generated for 16S rRNA gene and ITS1 libraries using the vegdist command of the vegan package v2.4–4 [55]. Non-metric multidimensional scaling (NDMS) was used to represent the distance matrix, with lower than to 0.2. Permutational multivariate analysis of variance (PERMANOVA) was performed using the adonis command (vegan) (9999 permutations). The Simper test was used to determine the genera that contributed most to differences in relative abundance at intra-site and inter-group levels. Relative abundances of main taxa were then compared with a Kruskal-Wallis test for the comparison of sampling conditions and a Mann-Whitney test for intra-site comparisons.

## Results

### Diversity of the microbial communities on earthen materials

A total of 72 samples collected at 12 sites were successfully amplified and sequenced. Three thousand five hundred forty-four fungal OTUs were identified across all ITS1 libraries and assigned to 495 fungal genera. For 16S rRNA gene bacterial sequences, 13,174 OTUs were detected, corresponding to 513 bacterial genera on all the samples. Among all samples, the OTU average was 171 (range: 28–571) for fungal communities and 1007 (range: 117–2682) for bacterial ones. For the dwelling sites, no difference of Shannon diversity index was observed between earthen materials with or without plant fibres, for either the fungal (mean: 2.84; range: 1.33–4.47) or the bacterial (mean: 4.57; range: 1.52–6.9) communities (Additional file 1: Figure S1). However, higher fungal (mean: 3.16; range: 3.06–3.34) and bacterial (mean: 5.26; range: 3.68–5.97) diversities were associated with materials containing vegetal aggregates at non-dwelling sites compared to raw-earth-only materials (fungi: 1.78; 1.61–2.10) (bacteria: 3.37; 1.52–4.52). The diversity of fungal and bacterial communities appeared to be correlated to each other (Spearman test *p*-value < 0.05; rho: 0.44), which means that sites with a high fungal diversity also contained a high bacterial diversity, and vice versa.

### Community structure of microbiome from earthen materials

**Fig. 1:**
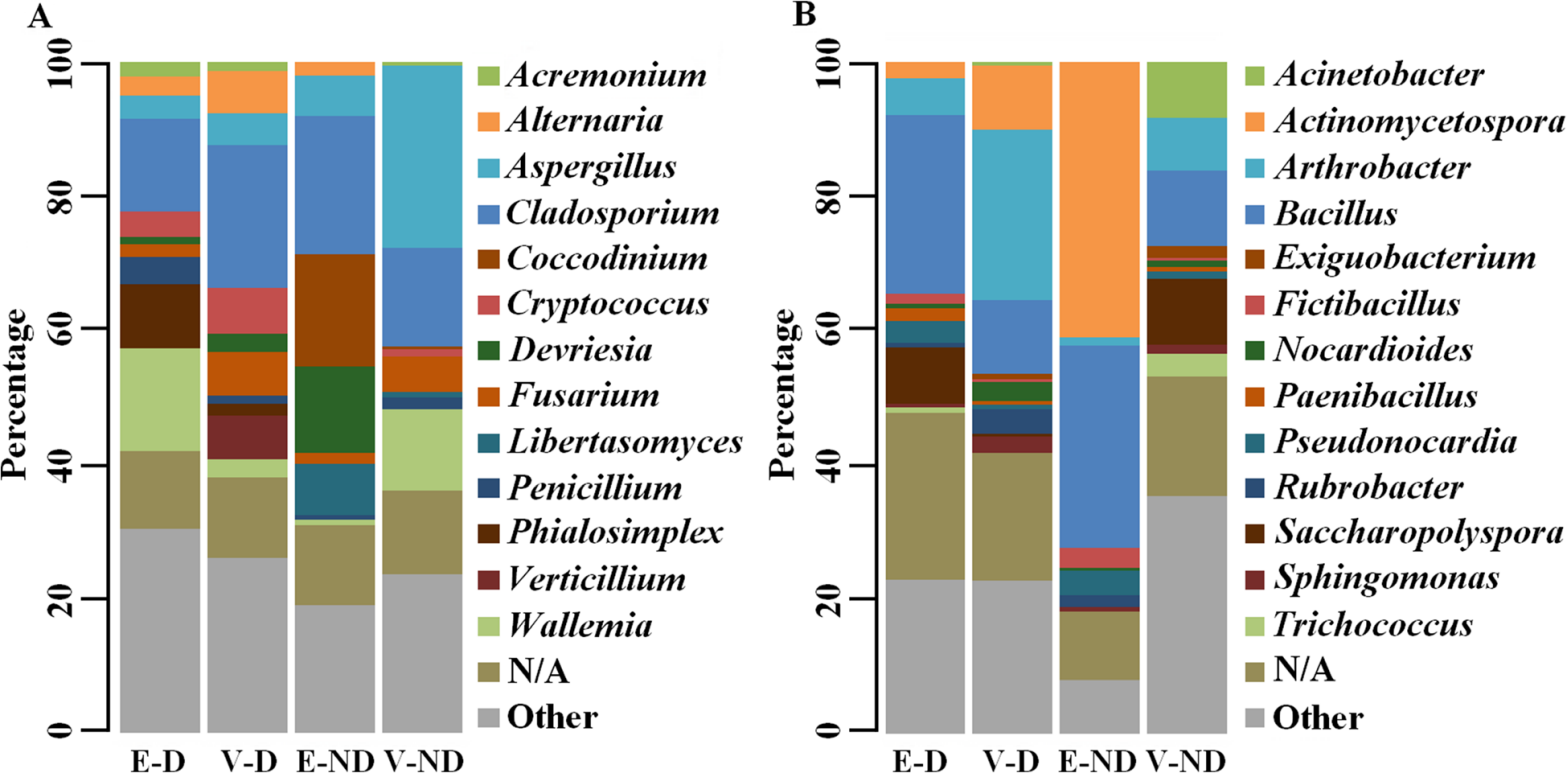
– Relative abundances of the major microbial taxa assigned by metabarcoding for the different locations and compositions. The relative abundances of the major fungal (A) and bacterial (B) taxa assigned are represented according to the different sample conditions: i) earthen materials without vegetal aggregates (E-D); ii) earthen materials with vegetal aggregates (V-D); iii) earthen materials without vegetal aggregates (E-ND); iv) earthen materials with vegetal aggregates (V-ND). The following taxa were significantly different among the four sampling conditions (Kruskal-Wallis *p*-value < 0.05): i) fungi – *Acremonium*, *Alternaria*, *Aspergillus*, *Cladosporium*, *Cryptococcus*, *Libertasomyces*, *Penicillium*, *Phialosimplex*, *Verticillium*, *Wallemia*; ii) bacteria – *Acinetobacter*, *Actinomycetospora*, *Arthrobacter*, *Exiguobacterium*, *Fictibacillus*, *Nocardioides, Saccharopolyspora*, *Sphingomonas*, *Trichococcus*. The following taxa were not significantly different among the four sampling conditions (Kruskal-Wallis p-value > 0.05): i) fungi – *Coccodinium*, *Devriesia*, *Fusarium*; ii) bacteria – *Bacillus*, *Paenibacillus*, *Pseudonocardia*, *Rubrobacter*.

The ITS1 sequences were mostly assigned to *Ascomycota* (76.51%) and *Basidiomycota* (18.59%), while 4.25% were unassigned and other phyla corresponded to less than 0.5% of the sequences. The main classes were *Dothideomycetes* (33.05%), *Eurotiomycetes* (20.71%), *Sordariomycetes* (15.52%), *Wallemiomycetes* (8.22%) and *Tremellomycetes* (5.36%). The most abundant taxa are reported in Fig. 1A. *Cladosporium* was the most widespread genus (15.53%), and was detected with some inter-site variability at all sampling sites. Genera detected at the majority of sites were *Wallemia* (8.21%), *Aspergillus* (8.18%), *Fusarium* (3.45%), *Cryptococcus* (3.61%) and *Alternaria* (3.18%). Other genera were prominently present at a few sites only, such as *Phialosimplex* (5.96%), *Devriesia* (2.35%), and *Verticillium* (1.97%). The culture-based approaches were conducted at 5 different sites demonstrating a low level of viable/culturable moulds at sites where there had been no hydric accident (< 10^2^ CFU/g, data not shown).

Regarding the bacterial flora, the major taxa were: *Actinobacteria* (39.61%), composed of *Actinobacteria* (39.61%) and *Rubrobacteria* (1.59%); *Firmicutes* (27.52%), composed of *Bacilli* (26.35%) and *Clostridia* (1.16%); *Proteobacteria* (9.97%), composed of *Alphaproteobacteria* (4.33%), *Gammaproteobacteria* (4.29%) and *Betaproteobacteria* (1.30%); *Bacteroidetes* (1.76%), composed of *Sphingobacteria* (0.93%) and *Flavobacteria* (0.61%). *Bacillus* (19.61%) and *Arthrobacter* (10.39%) were the predominantly detected genera (Fig. 1B). Some others were mainly detected at several sites, such as *Actinomycetospora* (7.95%) and *Saccharopolyspora* (6.96%). As detected with metabarcoding approaches, bacterial isolates (10^4^ to 10^6^ CFU/g, data not shown) from sites A to D mainly belonged to the genus *Bacillus*. MALDI-TOF mass spectrometry was used to define the diversity at the species level (See Additional file 1: Table S2). Other bacterial isolates were related to the soil environment (*Pseudomonas luteola* and *stutzeri*, *Solibacillus silvestris* and *Streptomyces griseus*) and potentially to the human microbiome (*Micrococcus luteus* and *Staphylococcus haemolyticus*). The predominance of Gram positive genera (87% versus 13% for Gram negative on assigned OTUs) was observed by metabarcoding across all the sampled buildings, leading to limited endotoxin risks. Moreover, almost no mycobacteria were detected in any of the samples.

### Factors impacting microbial communities on earthen materials

**Fig. 2:**
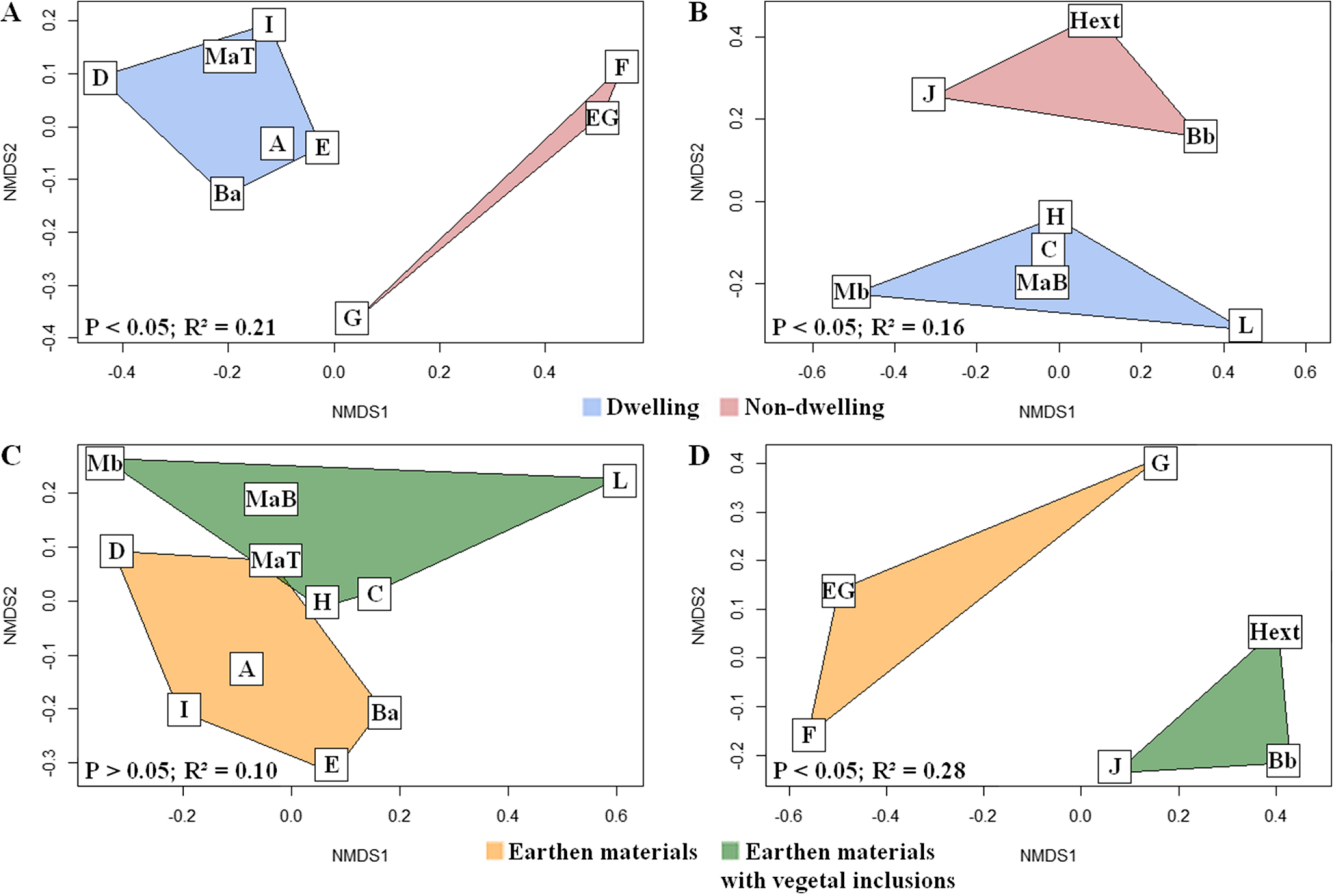
– NMDS representations of the Bray Curtis distance matrices for fungal communities. Bray Curtis distance matrices of fungal communities are represented using NMDS based on the different factors studied: i) dwellings vs non-dwellings, at E-D and E-ND sites (A) and V-D and V-ND sites (B); ii) earth-only materials vs earthen materials with vegetal aggregates, with E-D and V-D (C) and E-ND and V-ND (D). p-value and R^2^ of PERMANOVA test are indicated.

The distribution of fungal communities identified by metabarcoding was compared by NMDS between the two locations for the same wall composition, either on raw-earth-only materials (Fig. 2A) or earth materials with vegetal inclusions (Fig. 2B). The fungal profiles were significantly different, with a slightly more pronounced difference in the case of earthen-only supports (PERMANOVA *P* < 0.05, R^2^ = 0.21) compared to supports including vegetal aggregates (PERMANOVA *P* < 0.05, R^2^ = 0.16). When sites with different material compositions from the same location were compared, fungal communities sampled in dwellings were not significantly different (PERMANOVA *P* > 0.05) (Fig. 2C). In the inter-site comparison, the same composition at the dwelling sites led to similar overall relative abundances. However, some predominance may be noted as a greater abundance of *Wallemia* for sites E-D or *Fusarium* and *Verticillium* for sites V-D (Fig. 1). In contrast, samples from non-dwelling buildings (PERMANOVA *P* < 0.05, R^2^ = 0.28) (Fig. 2D) presented significant differences.

**Fig. 3:**
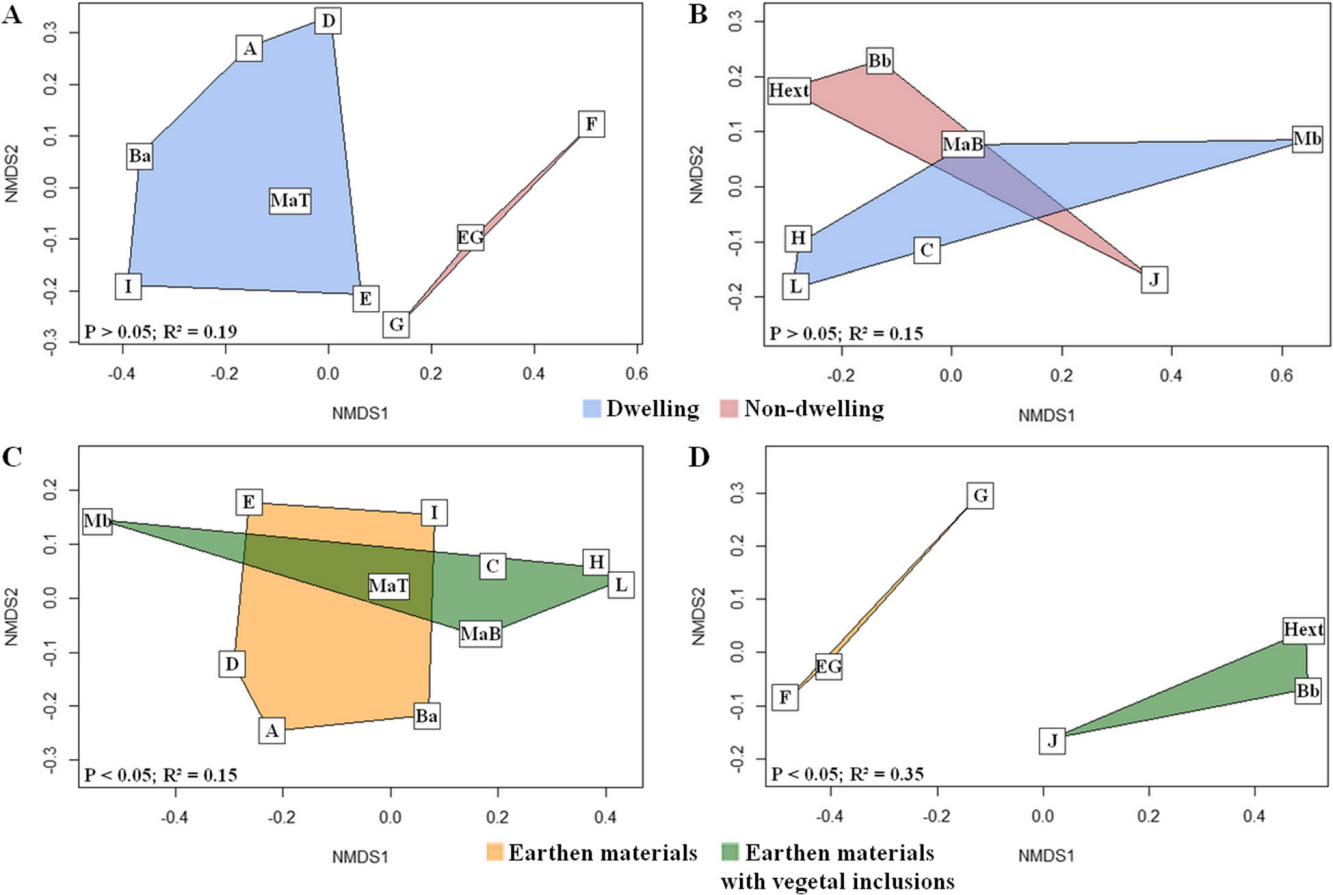
NMDS representations of the Bray Curtis distance matrices for bacterial communities. Bray Curtis distance matrices of fungal communities are represented using NMDS based on the different factors studied: i) dwellings vs non-dwellings, with E-D and E-ND sites (A) and V-D and V-ND sites (B); ii) earth-only materials vs earthen materials with vegetal aggregates, with E-D and V-D (C) and E-ND and V-ND (D). No significant effect was observed regarding the location condition. p-value and R^2^ of PERMANOVA test are indicated.

Concerning bacterial communities, no significant difference was observed when sites with the same conditions were compared according to the location (Fig. 3A & B; PERMANOVA *P* > 0.05). In contrast, differences were observed between sites in the same location conditions for support compositions of both the dwelling (Fig. 3C; PERMANOVA P < 0.05, R^2^ = 0.15) and the non-dwelling conditions (Fig. 3D; PERMANOVA P < 0.05, R^2^ = 0.35), with a stronger effect in the latter. Regarding the conditions of dwellings, some trends suggest an effect of the composition on the abundance of the genus *Arthrobacter*, which was more detected on earthen walls with plant fibres, while the genus *Bacillus* was more strongly associated with earth-alone materials. However, inter-site variability for the same composition and location conditions did not lead to any significant variation in genera (Mann-Whitney *p*-value > 0.05).

### Intra-site variability of microbial communities

Microbial communities were not found to be homogenous within samples from a given site (Additional file 1: Table S3). At site A, differences were observed between sample heights 190, 170, 150 and 70 cm on the one hand and 120 and 100 cm on the other hand. Some bacterial genera were significantly more abundant within the 120 cm and 100 cm samples than on the rest of the wall: the genera *Promicromonospora*, *Streptomyces*, *Arthrobacter* and *Paenisporosarcina* were almost absent from the 120–100 cm samples (relative abundance < 1.03%) whereas their relative abundance was higher than 1.8% on the rest of the wall (Mann-Whitney p-value < 0.05). Similarly, samples from the adobes at 160 and 110 cm at site D presented a different flora from that of the joints at the same heights and of the adobe and joint at 60 cm. The main part of the wall had a high abundance of *Saccharopolyspora* (47.52% ± 5.95%), *Prauserella* (10.45% ± 7.91%) and *Amycolatopsis* (8.45% ± 0.65%), whereas these genera were very scarce in adobe samples taken at 160 and 110 cm (relative abundance < 2%). This was counterbalanced by an abundance of *Bacillus* (34.43 ± 11.74%) and higher diversity. The samples also mainly contained *Phialosimplex* (94.76% ± 2.32%), indicating a probable development of this mould on the material, while the 160 and 110 cm adobes had a much more varied flora on their surface, including *Cladosporium* (23.28% ± 11.4%) and *Wallemia* (5.73% ± 6.75%). In addition, intra-site variations through time were evaluated with microbial communities analysed at sites sampled 1 year apart, and appeared to be highly similar over time (Additional file 1: Figure S2), suggesting a stability of the microbial community composition on earthen materials.

## Discussion

As soil is considered as one of the largest reservoirs of microbiological diversity [56], earthen materials are assumed to be colonized by a large biodiversity of microorganisms. To date, few works have been performed to describe the microbiome from such building materials, which have been used over hundreds of years and in which interest is currently reviving. To address this question, samples were taken within buildings without visual degradation in the aim of characterizing the bacterial and fungal communities present on the surface of earthen supports by using both metabarcoding and cultural approaches.

The observed predominance of *Ascomycota* (76.51%) in indoor communities was similar to results for samples from other housing, particularly in indoor dusts [28, 34]. Most major taxa detected, such as *Cladosporium*, *Aspergillus* or *Alternaria*, had been identified in previous studies on fungal flora in human habitats by using high-throughput sequencing [25, 28, 32, 34]. Regarding the bacterial flora, a prevalence of *Actinobacteria*, *Firmicutes* (mainly *Bacilli*) and *Proteobacteria* (mainly *α* and *Υ-proteobacteria*) was observed on earthen materials.

Almost all abundant fungal taxa were associated with the external environment, and also with the air (*Cladosporium*, *Aspergillus*, *Cryptococcus*, *Alternaria*, *Penicillium*, etc.) [32]. In contrast to air-associated fungal taxa, those related to the human microbiome (i.e. *Malassezia* sp.*, Rhodotorula* sp.) were detected at very low relative abundances. This prevalence of outdoor air floras relative to other types in the composition of fungal communities on indoor surfaces has already been reported [25]. Similarly to fungal taxa, almost all main bacterial genera detected (*Actinomycetospora*, *Arthrobacter*, *Bacillus*, *Nocardioides*, *Paenibacillus*, *Rubrobacter*, *Sphingomonas*) were ecologically associated with soil and the outdoor environment [57–60], and some of them were also identified in indoor environments, either in airborne particles and dust [30, 31] or on surfaces [26]. The culture methods were employed in order to confirm which genera were viable / culturable, and then which microorganisms may be able to proliferate in conditions of high humidity. Considering that identification at species level by 16S rRNA gene metabarcoding was not possible for some genera, especially for *Bacillus* [61, 62], identification of bacterial isolates was performed by MALDI-TOF mass spectrometry [63, 64]. Cultural results were consistent with the metabarcoding ones and confirmed the strong presence of soil related bacteria. This method also revealed a large species diversity of *Bacillus* across all samples. However, a bacterial flora on interior surfaces is usually associated with human flora [27], regarding surfaces in contact with occupants [26] or indoor air and dusts [30, 31, 65]. Lower physical contact with occupants than for other (horizontal) indoor surfaces is to be considered for walls, and literature about wall microflora is very sparse. Genera such as *Micrococcus* or *Staphylococcus* can be indicators of human flora but were detected very little on earthen supports (relative abundances < 1%). This low abundance of human-associated genera was also observed in culture, with the majority of isolates associated with the genus *Bacillus*. This particular bacterial community structure for an indoor surface could result from the presence of an initial high diversity of soil microorganisms, limiting bacterial colonization by microbial competition, combined with a potentially low ability of human-associated bacteria to grow on raw earth.

Based on the average profiles per dwelling, no significant impact of the presence of plant fibres in earthen materials was observed on the diversity of fungal communities. Despite the presence of plant fibres, which can increase the development of some fungal species able to degrade cellulose or lignin [7], these materials did not appear to induce a greater microbial diversity, either bacterial or fungal, under conventional environmental conditions. However, the genera *Fusarium* and *Verticillium* were more abundant at sites with plant aggregates. These two genera are particularly present in soils, including some phytopathogenic species [66, 67], and finally could have been brought in by the plant fibres during the manufacturing process. Their survival on the surface of the wall could have been promoted by vegetal aggregates. Nonetheless, the presence of plant fibres in earthen materials in a dwelling would not be a determining factor in the survival or development of specific fungal flora under standard conditions (excluding water damage). In contrast, non-dwelling buildings, exposed to outdoor environmental conditions, may present significant differences regarding the composition of the fungal communities on their surfaces, depending on the presence or absence of plant fibre in the material.

The metabarcoding analyses revealed intra site variation depending on the buildings studied. Some sites had more dispersed and variable communities within the different samples than other sites did, for both bacterial and fungal flora. This heterogeneity on the same wall can be explained by a particular use or by the history of the dwelling, such as water accidents or the use of the building over time (opened or closed premises, agricultural activity - such as the presence of animals or the storage of agricultural materials - bringing moisture and humidity, specific microorganisms, etc.) with a strong impact on microbial communities [13, 68, 69]. Of all the sites sampled in our study, only one presented traces of mould proliferation, which was due to a faulty drain several months before sampling. On this site, the particular abundance of the fungal genus *Wallemia* may have been a result of the previous high humidity accident, as past proliferations on building materials are observable despite remediation of the buildings [70]. In addition, many differences within microbial communities could be observed between different constructions. In the indoor environment, both fungal and bacterial floras were subject to many external factors inducing variations. Flora variations have previously been described regarding the geographical location [35] and the sampling period [25, 36, 38, 71]. Inter-site variation is a major challenge for the study of an interior flora, which often shows greater variability between buildings than between locations sampled within the same building [27, 38]. The incidence of such variations was not investigated in our study as we focused on a single campaign at 3 sites that differed in terms of geographical location and season. However, samplings 1 year apart performed on two sites suggested a certain stability of microbial communities on earthen materials over time for the same season, which could be investigated with new sampling campaigns across time. Another variable that could significantly influence the type of flora present in samples was the composition of the soil used as raw material. Several physical-chemical parameters have a direct effect on the structure of microbial communities present in soil [72] and possibly, by extension, in the final materials.

Considering health hazards, some of the abundant fungal genera detected belonged to potentially allergenic categories, such as the filamentous fungi *Cladosporium*, *Aspergillus*, *Fusarium*, *Alternaria* and *Penicillium* [73]. These genera are widely distributed in outdoor [32] and indoor environments [34] but do not generally have a detectable impact on the health of the occupants regarding the microbial load. A significant “unconventional” development of these microorganisms in a building (i.e. due to water damage) – generally with a visible development of mycelium and spores – can induce health issues [13, 14]. In addition, few endotoxin producers, which make up a large proportion of the bacteria involved in indoor air quality degradation [74], were detected in these supports. Most of the bacterial communities identified would therefore not imply specific risk to the occupants. The presence of this microbial diversity on earthen construction supports could have interesting effects on the indoor air quality compared to the case of conventional materials. A high density and diversity of microorganisms at the surface of earthen materials would prevent the colonization and development of other potentially harmful microorganisms, by occupying the ecological niche or competing against each other. Moreover, some studies have revealed that low microbial diversity in indoor dust implies higher risks of childhood asthma development [68, 75]. It is then tempting to hypothesize that the dense and diverse microbiome associated with earthen materials increases the safety of a dwelling environment by limiting potential invasive growth of pathogenic fungi and bacteria.

## Conclusion

Our study gives a first glimpse at the supposedly large diversity of the microbiome on raw earth materials, which were poorly characterized until now. We can already conclude that, although varied, the observed microbial diversity is lower than usually found in soil and is strongly associated with the outdoor and indoor environments. Bacterial communities, containing no specific harmful species and being less human-associated than communities in conventional building environments, seem to colonize these supports. Surprisingly, the inclusion of plant aggregates within earthen materials does not induce significant changes in the indoor fungal community structures. Regarding the design of the study, the major factors driving microbial diversity are the condition (indoor / outdoor) and the history of the materials (previous high humidity accident, storage of vegetables, etc.). These results highlight the possibility that the diversified and healthy microbiome found on earthen building materials could lower the risk of development of undesirable and harmful microorganisms.

## Supplementary information

**Additional file 1: Table S1.** Information about sampled buildings. Condition tags: E-D: earthen materials without vegetal aggregates – dwelling sites; V-D: earthen materials with vegetal aggregates – dwelling sites; E-ND: earthen materials without vegetal aggregates – non-dwelling sites; V-ND: earthen materials with vegetal aggregates – non-dwelling sites. Location tags: SW: south-west of France; E: east of France. **Table S2.** Bacterial isolates by culture from sites A to D as identified by MALDI-TOF mass spectrometry. **Table S3.** Microbial genera presenting a significantly different relative abundance at two parts of the same site. Relative abundances of bacterial and fungal genera listed in Additional file 1: Table S3 differed significantly (Mann-Whitney *p*-value < 0.05) depending on the sampling area within the same site. **Figure S1.** Shannon index for microbial communities sampled on earthen materials. The diversity of fungal (A) and bacterial (B) communities sampled on earthen materials was estimated using the Shannon index. *: Mann-Whitney test p-value < 0.05. **Figure S2.** Relative abundances of the major microbial taxa assigned for two sites sampled 1 year apart. The relative abundances of the major fungal (A) and bacterial (B) taxa assigned for sites Ba and C are compared between two samples collected 1 year apart (T0 and T1). The following taxa significantly differed between the two sampling times (Mann-Whitney p-value < 0.05): i) fungi – Ba: *Fomes*, *Inonotus*, *Scedosporium*, *Stemphylium*; C: Ø. ii) bacteria – Ba: *Skermanella*; C: Ø.

## Data Availability

The datasets generated and analysed during the current study are available in the PRJNA542045 repository, https://www.ncbi.nlm.nih.gov/sra/PRJNA542045.

## Abbreviations

E-D: Earthen materials without vegetal aggregates in dwelling sites
E-ND: Earthen materials without vegetal aggregates in non-dwelling sites
ITS: Internal transcribed spacer
NMDS: Non-metric multidimensional scaling
OTU: Operational taxonomic unit
PERMANOVA: Permutational multivariate analysis of variance
RH: Relative humidity
TSA: Tryptone soy agar
V-D: Earthen materials with vegetal aggregates in dwelling sites
V-ND: Earthen materials with vegetal aggregates in non-dwelling sites
