# Supplementary material for: Characterization of the microbiome associated with in situ earthen materials
Source: Environ Microbiome. 2020 Jan 20;15:4. doi: 10.1186/s40793-019-0350-6 (PMC8066951; doi:10.1186/s40793-019-0350-6)
Supplement: Supplementary file 1 — Additional file 1: Table S1. Information about sampled buildings. Condition tags: E-D: earthen materials without vegetal aggregates – dwelling sites; V-D: earthen materials with vegetal aggregates – dwelling sites; E-ND: earthen materials without vegetal aggregates – non-dwelling sites; V-ND: earthen materials with vegetal aggregates – non-dwelling sites. Location tags: SW: south-west of France; E: east of France. Table S2. Bacterial isolates by culture from sites A to D as identified by MALDI-TOF mass spectrometry. Table S3. Microbial genera presenting a significantly different relative abundance at two parts of the same site. Relative abundances of bacterial and fungal genera listed in Additional file 1: Table S3 differed significantly (Mann-Whitney p-value < 0.05) depending on the sampling area within the same site. Figure S1. Shannon index for microbial communities sampled on earthen materials. The diversity of fungal (A) and bacterial (B) communities sampled on earthen materials was estimated using the Shannon index. *: Mann-Whitney test p-value < 0.05. Figure S2. Relative abundances of the major microbial taxa assigned for two sites sampled 1 year apart. The relative abundances of the major fungal (A) and bacterial (B) taxa assigned for sites Ba and C are compared between two samples collected 1 year apart (T0 and T1). The following taxa significantly differed between the two sampling times (Mann-Whitney p-value < 0.05): i) fungi – Ba: Fomes, Inonotus, Scedosporium, Stemphylium; C: Ø. ii) bacteria – Ba: Skermanella; C: Ø. [file 40793_2019_350_MOESM1_ESM.docx]

Table S1 - Information about sampled buildings.

| **Conditions** | **Sampling label** | **Type of materials** | **Function** | **Date of building** | **History** | **Location** | **Sampling month** | **Number of samples** | | |
| --- | --- | --- | --- | --- | --- | --- | --- | --- | --- | --- |
|  |  |  |  |  |  |  |  | **16S rRNA gene** | **ITS1 regions** | |
| E-D | AM | Raw earth adobe | Hall | Before 1800 | Previously a barn housing farm animals | Maureville (SW) | September 2015 | 6 | | 6 |
|  | AJ | Joint (raw earth + lime) |  |  |  |  |  | 6 | | 6 |
|  | Ba1 | Raw earth adobe | Hall | August 1995 | Previous fungal development caused by a blocked drain | Castanet-Tolosan  (SW) | October 2015 | 2 | | 2 |
|  | Ba2 |  |  |  |  |  | November 2015 | 4 | | 4 |
|  | DM | Raw earth adobe | Kitchen | Unknown | Probable presence of a plaster on the surface of the wall | Maureville  (SW) | November 2015 | 3 | | 3 |
|  | DJ |  |  |  |  |  |  | 3 | | 3 |
|  | E | Rammed earth | Bedroom | Around 1800 | Dwelling | Tignieu-Jameyzieu (E) | July 2016 | 5 | | 5 |
|  | I | Raw earth adobe | Living room | July 2008 | Dwelling | Mirabel  (SW) | November 2016 | 4 | | 4 |
|  | MaT | Raw earth adobe | Hall | Summer 2016 | Dwelling | Caussade  (SW) | January 2017 | 2 | | 1 |
| V-D | C1 | Plaster (raw earth + hemp) | Office | Spring 2009 | Dwelling | Lanta  (SW) | November 2015 | 4 | | 4 |
|  | C2 |  |  |  |  |  | November 2016 | 4 | | 4 |
|  | H | Plaster (raw earth + straw) | Office | 2013 | Dwelling | L'Union  (SW) | October 2016 | 4 | | 4 |
|  | L |  | Kitchen | 2006 | Dwelling | Cazals  (SW) | November 2016 | 3 | | 2 |
|  | MaB | Adobe (raw earth + straw) | Hall | Summer 2016 | Dwelling | Caussade  (SW) | January 2017 | 3 | | 3 |
|  | Mb | Adobe (raw earth + straw) | Bedroom | 2012 | Dwelling | Caussade  (SW) | January 2017 | 4 | | 4 |

| **Conditions** | **Sampling label** | **Type of materials** | **Function** | **Date of building** | **History** | **Location** | **Sampling month** | **Number of samples** | | |
| --- | --- | --- | --- | --- | --- | --- | --- | --- | --- | --- |
|  |  |  |  |  |  |  |  | **16S rRNA gene** | **ITS1 regions** | |
| E-ND | EG | Rammed earth | Barn | Around 1800 | Storage room | Tignieu-Jameyzieu  (E) | July 2016 | 1 | | 1 |
|  | F | Rammed earth | Barn | Around 1800 | No closed doors or windows | Tignieu-Jameyzieu  (E) | July 2016 | 4 | | 4 |
|  | G | Rammed earth | Cellar | Around 1800 | Storage room | Tignieu-Jameyzieu  (E) | July 2016 | 4 | | 4 |
| V-ND | Bb | Cob (raw earth + straw) | Cellar | August 1995 | Regular storage of fruit and vegetables | Castanet-Tolosan  (SW) | October 2015 | 5 | | 5 |
|  | Hext | Adobe (raw earth + straw) | Outdoor material | 2013 | Material left outside since construction | L'Union  (SW) | October 2016 | 1 | | 1 |
|  | J | Plaster (raw earth + vegetal fibres) | Non-dwelt room | Before 1900 | No occupant and low insulation | Monteils  (SW) | November 2016 | 4 | | 4 |

Condition tags: E-D: earthen materials without vegetal aggregates – dwelling sites; V-D: earthen materials with vegetal aggregates – dwelling sites; E-ND: earthen materials without vegetal aggregates – non-dwelling sites; V-ND: earthen materials with vegetal aggregates – non-dwelling sites. Location tags: SW: south-west of France; E: east of France

Table S2 – Bacterial isolates by culture from sites A to D as identified by MALDI-TOF mass spectrometry.

|  |  | Site | | | | |
| --- | --- | --- | --- | --- | --- | --- |
| Taxa | | A | Ba | D | C | Bb |
| *Bacillus* | *atrophaeus* |  |  |  |  | • |
|  | *cereus* |  | • |  |  |  |
|  | *indicus* |  |  | • |  |  |
|  | *licheniformis* | • |  | • |  |  |
|  | *mojavensis* | • |  |  |  |  |
|  | *megaterium* |  |  | • |  | • |
|  | *muralis* |  | • | • |  |  |
|  | *mycoides* |  |  |  |  | • |
|  | *niacini* |  |  |  |  | • |
|  | *pseudomycoides* |  |  |  | • | • |
|  | *pumilus* |  | • |  | • |  |
|  | *simplex* | • | • | • | • | • |
|  | *subtilis* | • |  |  |  |  |
| *Micrococcus* | *luteus* |  |  |  | • |  |
| *Pseudomonas* | *luteola* | • |  |  |  |  |
|  | *stutzeri* |  | • |  |  |  |
| *Solibacillus* | *silvestris* |  |  |  |  | • |
| *Staphylococcus* | *haemolyticus* | • |  |  |  |  |
| *Streptomyces* | *griseus* |  |  |  | • |  |

Table S3 - Microbial genera presenting a significantly different relative abundance at two parts of the same site.

|  |  | Relative abundance means ± standard deviation | | | |
| --- | --- | --- | --- | --- | --- |
|  |  | **Bacterial genera** | | | |
| Site | Sampling areas | *Arthrobacter* | *Paenisporosarcina* | *Promicromonospora* | *Streptomyces* |
| A | 190 - 150 cm + 70 cm | 0.87 ± 0.66 % | 0.06 ± 0.05 % | 0.11 ± 0.14 % | 0.41 ± 0.41 % |
|  | 120 - 100 cm | 3.23 ± 1,76 % | 1.89 ± 0.92 % | 20.31 ± 10.15 % | 10.35 ± 6.63 % |
|  |  | *Amycolatopsis* | *Bacillus* | *Prauserella* | *Saccharopolyspora* |
| D | 160 - 110 cm (joint) + 60 cm (adobe & joint) | 8.45 ± 0.65 % | 1.53 ± 0.59 % | 10.45 ± 7.91 % | 47.52 ± 5.95 % |
|  | 160 - 110 cm (adobe) | 0.04 ± 0.07 % | 34.43 ± 11.74 % | 0.06 ± 0.01 % | 1.67 ± 1.90 % |
|  |  | **Fungal genera** | | | |
|  |  | *Cladosporium* | *Cryptococcus* | *Phialosimplex* | *Wallemia* |
| D | 160 - 110 cm (joint) + 60 cm (adobe & joint) | 0.45 ± 0.35 % | 0.36 ± 0.23 % | 94.76 ± 2.32 % | 0.44 ± 0.33 % |
|  | 160 - 110 cm (adobe) | 23.28 ± 11.40 % | 19.98 ± 23.08 % | 0.25 ± 0.16 % | 5.73 ± 6.75 % |

Relative abundances of bacterial and fungal genera listed in Supplementary Table 3 differed significantly (Mann-Whitney p-value < 0.05) depending on the sampling area within the same site.


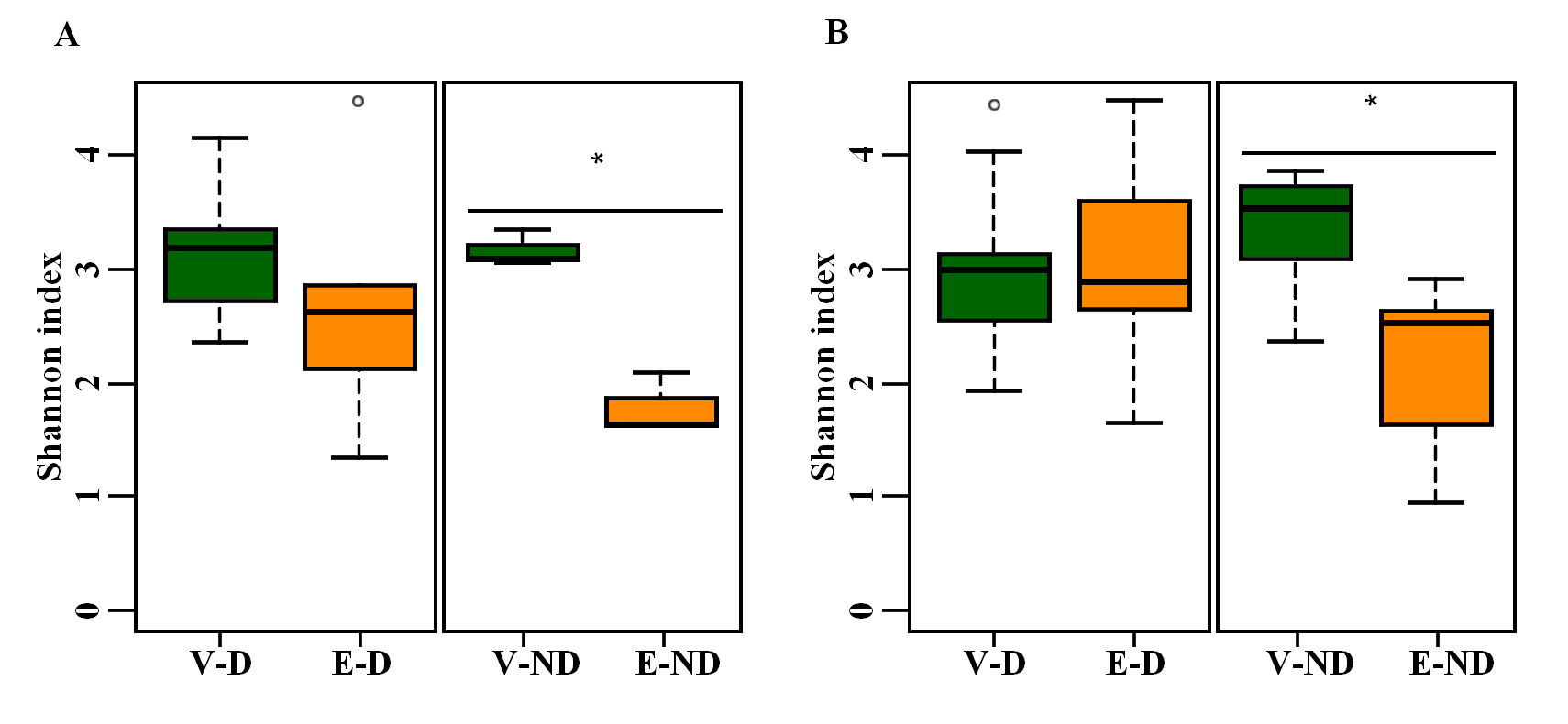


Figure S1 – Shannon index for microbial communities sampled on earthen materials.

The diversity of fungal (A) and bacterial (B) communities sampled on earthen materials was estimated using the Shannon index. *: Mann-Whitney test p-value < 0.05.


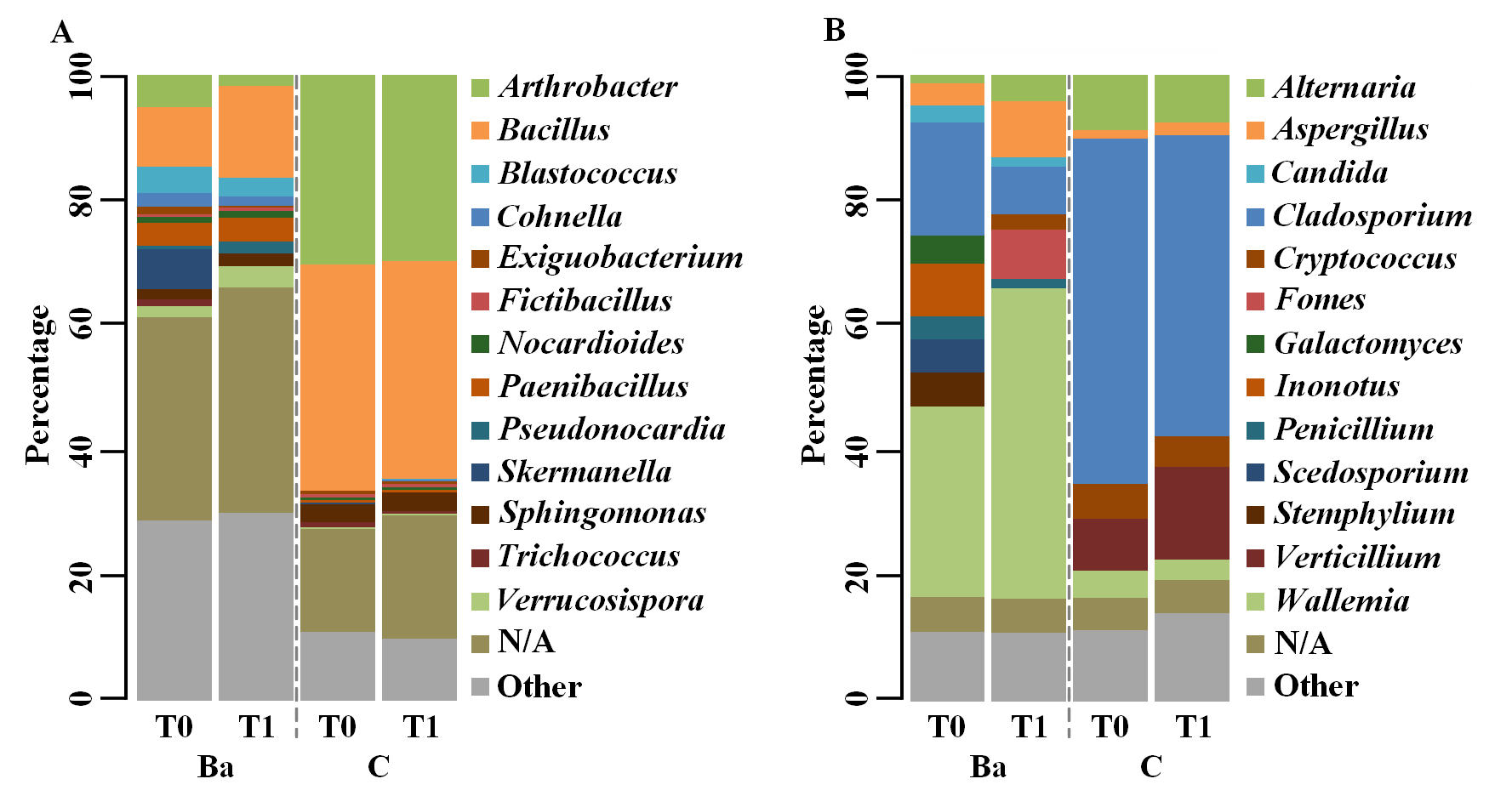


Figure S2 – Relative abundances of the major microbial taxa assigned for two sites sampled one year apart.

The relative abundances of the major fungal (A) and bacterial (B) taxa assigned for sites Ba and C are compared between two samples collected one year apart (T0 and T1). The following taxa significantly differed between the two sampling times (Mann-Whitney p-value < 0.05): i) fungi – Ba: *Fomes*, *Inonotus*, *Scedosporium*, *Stemphylium*; C: Ø. ii) bacteria – Ba: *Skermanella*; C: Ø.
